# Real-time prediction and adaptive adjustment of continuous casting based on deep learning

**DOI:** 10.1038/s44172-023-00084-1

**Published:** 2023-06-07

**Authors:** Ziqing Lu, Neng Ren, Xiaowei Xu, Jun Li, Chinnapat Panwisawas, Mingxu Xia, Hongbiao Dong, Eric Tsang, Jianguo Li

**Affiliations:** 1 Shanghai Key Laboratory of Advanced High-temperature Materials and Precision Forming, School of Material Science and Engineering, Shanghai Jiao Tong University, 200240 Shanghai, China; 2 School of Engineering and Materials Science, Queen Mary University of London, London, E1 4NS UK; 3 School of Engineering, University of Leicester, Leicester, LE1 7RH UK; 4 School of Computer Science and Engineering, Macau University of Science and Technology, Taipa, Macau China

**Keywords:** Engineering, Techniques and instrumentation, Computational science, Metals and alloys

## Abstract

Digitalisation of metallurgical manufacturing, especially technological continuous casting using numerical models of heat and mass transfer and subsequent solidification has been developed to achieve high manufacturing efficiency with minimum defects and hence low scrappage. It is still challenging to perform adaptive closed-loop process adjustment using high-fidelity computation in real-time. To address this challenge, surrogate models are a good option to replace the high-fidelity model, with acceptable accuracy and less computational time and cost. Based on deep learning technology, here we developed a real-time prediction (ReP) model to predict the three-dimensional (3D) temperature field distribution in continuous casting on millisecond timescale, with mean absolute error (MAE) of 4.19 K and mean absolute percent error (MAPE) of 0.49% on test data. Moreover, by combining the ReP model with machine learning technology—Bayesian optimisation, we realised the rapid decision-making intelligent adaptation of the operating parameters for continuous casting with high predictive capability. This innovative and reliable method has a great potential in the intelligent control of the metallurgical manufacturing process.

## Abstract (web summary)

Ziqing Lu and colleagues employ deep learning to establish real-time predictions of three-dimensional temperature fields at the millimetre scale during continuous casting, a process widely used in metallurgical manufacturing. The researchers go on to combine the model with Bayesian optimisation for intelligent adaptation of operating parameters to fulfil various industrial production demands.

## Introduction

Continuous casting (CC) is a sophisticated metallurgical process used to manufacture most of the technological steel products (including billets, blooms, and slabs) around the world. There are always several types of casting defects including surface and corner cracks, centre macrosegregation, centre shrinkage, and porosity in the CC billets, which reduced the working performance of the steel products. An effective solution to overcome these defects is to use soft reduction technology, in which the key technical parameters are the position and amount of reduction, and appropriate secondary cooling water control. For the effective control of the secondary cooling and the soft reduction, the thickness of the solidified shell and the metallurgical length (the distance where solidification is sufficiently complete in slab centre) in the CC process need to be accurately estimated^1^. Therefore, it is of great importance to analyse altogether the heat transfer, solidification, multiphase turbulent flow, and other interacting phenomena in the CC process. However, it is rather difficult to perform enough experiments on CC process to generate data for digitalisation of the process, and due to the limitation of measurement techniques and harsh production environment, most of the important information in CC processes cannot be obtained by direct experiment. Numerical simulation is widely applicable to gain a better understanding on these fundamental behaviours, and subsequently predict the 3D temperature distribution and solidification shell, which are key information for process optimisation and defect mitigation strategy.

Direct numerical simulation (DNS) model based upon computational fluid dynamics (CFD) calculation for CC has greatly improved since the 1980s^2^, and has been used to optimise the operating conditions, such as the cooling water arrangement in the secondary cooling zone, resulting in the reduction of casting defects in the strand. The DNS models have been developed to simulate the complex phenomena during the CC process^3–6^, and studies have combined the DNS with optimisation-regulation algorithms^7–10^, in order to speed up the optimisation process for a more appropriate arrangement of cooling water. However, a common problem of these models is the excessive computational time and resource consumption, especially in parametric studies for the process optimisations where a great number of DNS calculations need to be performed, even in serial. Besides, there are always situations where the actual process behaviours misbehave an unexpected way. In this case, a fast decision and an autocorrect response are intensely needed to prevent the process from becoming more exacerbated.

Deep learning (DL) has emerged as a powerful technology, exhibiting state-of-the-art performance on a variety of tasks. With its exceptional ability to learn from vast amounts of data, DL techniques has been widely used in CC process^11^ to achieve continuous monitoring (3D laser image scanning system based on binocular imaging and DL techniques to detect, recognise, classify, and delineate the defects in CC product surfaces^12^), control (temperature control optimisation^13^ and molten steel temperature preset^14^ in the CC process with deep neural networks.), and assessment of the implementation (internal crack prediction^15^ and breakout prediction^16^ in the CC process with deep neural networks). Despite the benefits that DL techniques have offered for improving the intelligence and efficiency of the CC process, there is still a pressing need to develop methods that enable fast decision-making and rapid autocorrection response in this field.

For real-time monitoring and fast response of heat transfer and solidification phenomena in the CC process, by simplifying the numerical model^17,18^ and enhancing computing resources^19–21^ could help speed up the process optimisation; still these approaches have to compromise the accuracy and/or require unacceptable computing resources. Whereas the conventional DNS technology cannot offer real-time digital representation considering the demanding computational time, resources and accuracy. To circumvent the limitations of conventional DNS techniques, researchers have attempted to develop surrogate models using DL techniques to predict the DNS results with acceptable accuracy and less computational time and cost. For instance, CNNs-based autoencoder has been utilised to predict CFD velocity field by signed distance function^22,23^. To deal with irregular geometry grid, fully connected neural networks has been employed to predict the temperature evolutions calculated by finite element models^24^. In order to establish a mapping from the parametric space of the problem to its solution space, Nikolopoulos et al. applied a CNNs-based autoencoder and a feed-forward neural network to efficiently map points from the parametric space to the compressed version of the respective solution matrices^25^. Since the DNS results are highly related to the corresponding technological parameter setting, the DL model can be used to learn the relationships between them, so as to achieve rapid prediction of DNS results under the corresponding parameter settings. However, for different DNS models and varied prediction needs, building the corresponding dataset is challenging and resource-intensive, and more efforts are required to select and establish the appropriate DL models to build the corresponding surrogate models. Although DL-based surrogate model for CC has been reported^26^, which incorporated CNNs and recurrent neural networks to address both spatial and sequential information, it is limited to a 2D simulation prediction and focuses more on time series prediction.

In this study, we established an efficient surrogate model for a 3D CC heat transfer DNS process and demonstrated its potential industrial application. The proposed real-time prediction (ReP) model is capable of computing the 3D temperature fields of CC process with the aid of DL techniques, and combining with Bayesian optimisation (BO) to conduct the intelligent adaptation, it can improve the key operating conditions to achieve the expected target. The difference and contribution between our study and prior work are listed as followed: (1) Different tasks: Our surrogate model aims to predict the 3D temperature field during CC process under different casting speed, cooling water flow rate. (2) Different approaches: For our specific data form and task requirements, we have designed a specific model structure to achieve the best performance. (3) Exploration of application prospect of surrogate model: We aim to demonstrate how the surrogate model can be applied to process decision making and, in an attempt, to serve real-world metallurgical manufacturing engineering to meet the needs of intelligent control. We first developed a combined hybrid 3D/2D model^3^ to produce the CC DNS data. With the established 800 dataset of 3D simulated temperature fields (700 for training and 100 for testing), we trained a convolutional neural network (CNN)-based autoencoder^27^ to extract the latent code from the data, and reconstructed the data using the latent code. Then we developed an MLP-Mixer-based^28^ parameter encoder to map the technological parameter setting (casting speed and cooling water flow rate in eight different cooling zone) to the corresponding latent code. The ReP model can rapidly predict the 3D temperature fields accurately on millisecond timescale, without the requirement for excessive resources.

## Results

### Model construction

We hypothesise that the CC DNS temperature field (*T*_f_) is a complex function *F* of the corresponding technological parameter setting (*p*), as shown Eq. 1.

1 $${T}_{{{{{{\rm{f}}}}}}}=F\left(p\right)$$

**Fig. 1:**
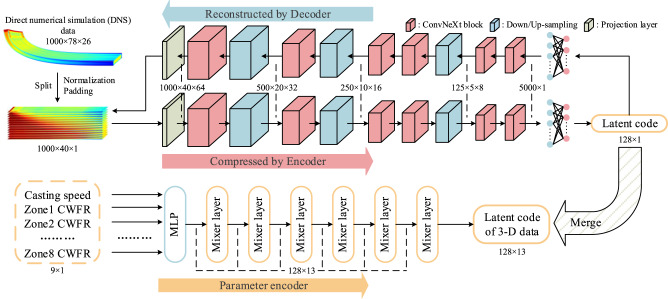
Framework of the real-time prediction model. The 1000×78×26 simulated temperature field from computational fluid dynamic (CFD) calculation was split by layers, and then normalised and padded to thirteen 1000×40×1 shaped data to train the encoder-decoder structure autoencoder. The latent coder extracted by the encoder part was merged to a 128×13 latent code of 3D dataset, to train the parameter encoder to map the technological parameter setting (casting speed and cooling water flow rate (CWFR) in each cooling zone) onto the corresponding latent code.

A real-time prediction (ReP) model has been successfully developed to fit function *F* to predict the temperature field under the corresponding technological parameter setting. The process is divided into two parts: encoder-decoder structure autoencoder and parameter encoder, as shown in Fig. 1. To extract the most valuable featured information from the CC data, we trained a self-supervision CNN-based autoencoder with the DNS data as the input and the output; the autoencoder needs to compress the 3D DNS data to a one-dimensional 128×1 latent code, and reconstruct the DNS data, so the autoencoder can extract the essential information from the data by the encoder part, and reconstruct the data by the decoder part using Eq. 2 and Eq. 3, where *l*_c_, *T*_f_, ${T}_{{{{{{\rm{f}}}}}}}^{{\prime} }$, *E*, *D* represent latent code, DNS temperature field, reconstructed temperature filed, encoder and decoder, respectively.

2 $${l}_{{{{{{\rm{c}}}}}}}=E\left({T}_{{{{{{\rm{f}}}}}}}\right)$$

3 $${T}_{{{{{{\rm{f}}}}}}}^{{\prime} }=D\left({l}_{{{{{{\rm{c}}}}}}}\right)$$

The Multilayer Perception (MLP) layer placed at the end of the encoder and the beginning of the decoder works as a nonlinear projection head that produces the latent code, which can improve the representation quality^29^. Considering symmetry and computational cost, we only take a quarter of the 1000×78×26 3D domain (20362×1530×190 mm^3^), then split it by layers, and zero pad it into thirteen 1000×40×1 data for training, as shown in Fig. 1.

With the well-trained autoencoder, then we trained a parameter encoder to map the technological parameter sets to the corresponding latent code. As our autoencoder extracts thirteen layers of 128×1 latent codes for each 3D data points, the autoencoder can only ‘see’ one layer of the 3D data at a time. As a result, the information between layers in the 3D temperature field is completely ignored. Since the temperature between two layers is interrelated, we consider this information in the parameter encoder part. Therefore, we employed an MLP-Mixer-based parameter encoder to map the 9×1 technological parameter setting to the corresponding latent code (128×13) of 3D data, as shown in Eq. 4, where ${l}_{{{{{{\rm{c}}}}}}}^{{\prime} }$, *p*, *P* are the latent code prediction, technological parameter setting and the parameter encoder, respectively. We proved the effectiveness in this approach by reintroducing the information between layers in the parameter encoder part (see Supplementary Fig. 4).

4 $${l}_{{{{{{\rm{c}}}}}}}^{{\prime} }=P\left(p\right)$$

The structures of autoencoder and parameter encoder are selected after experimental verification (see Supplementary Figs. 3–5) to ensure the best performance. The loss function and evaluation metric are described with mean absolute error (MAE) as Eq. 5 and mean absolute percent error (MAPE) as Eq. 6, where ${T}_{i,x,y,z}$, ${T}_{i,x,y,z}^{{\prime} }$ and $n$ are ground truth values, predicted values and total sample number.

5 $${{{{{\rm{MAE}}}}}}\left(T,{T}^{{\prime} }\right)=\frac{1}{n}\frac{1}{1000\times 39\times 13}{\sum }_{i=1}^{n}{\sum }_{x=1}^{1000}{\sum }_{y=1}^{39}{\sum }_{z=1}^{13}\left|{T}_{i,x,y,z}-{T}_{i,x,y,z}^{{\prime} }\right|$$

6 $${{{{{\rm{MAPE}}}}}}\left(T,{T}^{{\prime} }\right)=\frac{1}{n}\frac{1}{1000\times 39\times 13}{\sum }_{i=1}^{n}{\sum }_{x=1}^{1000}{\sum }_{y=1}^{39}{\sum }_{z=1}^{13}\left|\frac{{T}_{i,x,y,z}-{T}_{i,x,y,z}^{{\prime} }}{{T}_{i,x,y,z}}\right|$$

### Real-time prediction results

With the well-trained autoencoder and parameter encoder models, we can complete the 3D temperature field prediction, as shown in Eq. 2(a) and Eq. 7, where ${T}_{{{{{{\rm{f}}}}}}}^{{{{{{\rm{{{\hbox{'}}}}}}}{{\hbox{'}}}}}}$, *p*, *D*, *P* are the temperature field prediction, technological parameter setting, decoder and parameter encoder, respectively. The 9×1 technological parameter setting is first encoded into 128×13 latent code by the parameter encoder, then the decoder will decode the latent code into temperature field prediction.

7 $${T}_{{{{{{\rm{f}}}}}}}^{{{{{{\rm{{{\hbox{'}}}}}}}{{\hbox{'}}}}}}=D\left(P\left(p\right)\right)$$

**Fig. 2:**
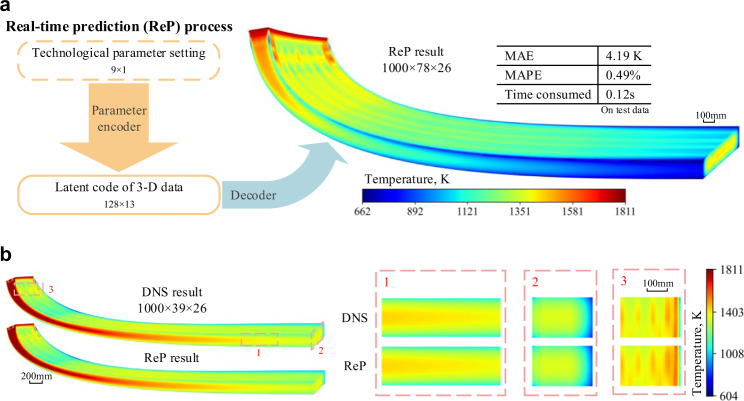
Workflow and results of the real-time prediction (ReP) model. **a** Workflow of the real-time prediction model. The parameter encoder map the 9×1 technological parameter set to the corresponding 128×13 latent code of 3D data, then the decoder part of the autoencoder predicts the 1000×78×26 3D temperature field by the latent code. It takes only 0.12 s for the real-time prediction model to complete. The mean absolute error (MAE) and mean absolute percent error (MAPE) on the test data are 4.19 K and 0.49%, respectively. **b** Comparison between 3D temperature fields obtained by ReP Model and direct numerical simulation (DNS). Half of the slab is shown, and three sub-regions are highlighted.

A typical 3D temperature field predicted by ReP model is shown in Fig. 2a. In order to verify the overall reliability of the ReP model, we compared the 3D temperature fields predicted by ReP model and DNS respectively, as shown in Fig. 2b. The predicted results by ReP model are in good agreement with the DNS results.

**Fig. 3:**
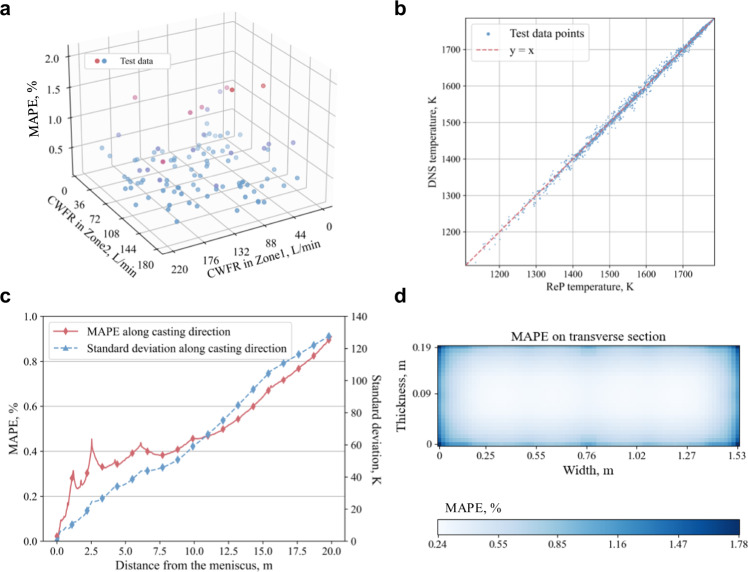
Accuracy analysis of ReP model. **a** Mean absolute percent error (MAPE) distribution of 100 test data points, with cooling water flow rate (CWFR) in cooling zone 1 and cooling zone 2 as the x-axis and y-axis. Blue points represent MAPE values less than 1.0% and include 88 data points, with 68 data points have MAPE values less than 0.5% and 20 data points have MAPE values between 0.5% and 1.0%. Red points indicate MAPE values between 1.0% and 2.1%, with 10 data points having MAPE values between 1.0% and 1.5% and 2 data points having MAPE values between 1.5% and 2.1%. **b** Plot of ReP temperature and DNS temperature of selected test data points. Blue points represent the predicated temperature, red dashed line corresponds to the predicted value being the same as the DNS temperature. **c** MAPE and standard deviation distribution along casting direction in test data. Red curve shows the average MAPE on all test data at different distances from the meniscus, and blue dashed curve represent the standard deviation of the temperature at different distance in all the test data. **d** MAPE distribution on the transverse section in test data.

The MAPE result of test data can better reflect the performance of the model, since the test data is isolated from the training process. Therefore, to demonstrate the performance of the model, we mainly use the MAPE result of the test data for the verification. In Fig. 3a, the MAPE distribution of the ReP model on 100 test data is plotted. First, we ensure the uniformity of data points by sampling algorithms for the accuracy and robustness of our model. The MAPE is very low on most of the test data (blue points, MAPE ≤ 0.5%: 68, 0.5 < MAPE ≤ 1.0%: 20). Though the error is a bit higher on a small group of test data (red point, 1.0% <MAPE ≤ 1.5%: 10, 1.5% <MAPE ≤ 2.1%: 2), these data is clustered at the origin and corresponding to the very low cooling water flow rate (CWFR), which is rarely used in industry.

To show the accuracy of the ReP model at each data point more deeply, we plotted the ReP temperature and DNS temperature results in Fig. 3b. However, because the total number of the test data points is too large (100 test data, each contains 507,000 (1000×39×13) data points), we uniformly selected 27 points from each test data (in each 1000×39×13 test data, selected points at 100, 500, 900 in the first dimension, 10, 20, 30 in the second dimension, 1, 5, 9 in the third dimension), 2700 data points in total. As shown in Fig. 3b, the ReP temperature results fits the DNS temperature results very well, with *R*^2^ score of 0.9982. And the *R*^2^ score between the ReP and DNS results on the whole test data point is 0.9987.

Furthermore, we demonstrate the accuracy of the ReP model in two different dimensions: along casting direction (Fig. 3c) and on the transverse section (Fig. 3d). We calculated the average MAPE at a certain location or section over all the test data to analyse the accuracy of the model in depth. In Fig. 3c, the solid red curve shows the average MAPE on all test data at different distances from the meniscus. The value of MAPE starts nearly zero at the beginning and then increases with the distance from the meniscus, reaching 0.91% at the bottom of the slab. We believe that this phenomenon is related to the complexity of the temperature fields in the data. For example, since in our case the temperature at the beginning of the slab (the mould) is affected only by the casting speed under the same pouring temperature, the ReP model can learn this relationship easily. As the distance from the meniscus increases, the temperature is influenced by more and more factors, the casting speed, the CWFR in the current cooling zone and the previous zone. It is therefore getting harder for the ReP model to make an accurate prediction. To demonstrate this, we calculated the standard deviation of the temperature at different distance in all the test data, as illustrated in the blue curve of Fig. 3c. It can be inferred that the non-uniformity distribution of the error is consistent with the standard deviation, which is confirmed by the similarity of the error and standard deviation distribution along the casting direction. Similarly, the average MAPE is calculated on all test data on the transverse section, as shown in Fig. 3d. The MAPE on the surfaces is relatively larger, especially on the lateral surfaces where it reaches the maximum value of 1.78%; in the interior of the slab, it is very small, value of which ranges from 0.24% to 0.6%. Overall, by analysing the errors in two different dimensions, it can be concluded that the errors of the prediction by the ReP model are relatively small, this has confirmed further the validated reliability of the model.

The ReP model can effectively make real-time prediction on the 3D temperature field of the continuous casting process within only 0.12 s (on personal laptop with CPU: AMD Ryzen 7 5800H and GPU: NVIDIA GeForce RTX 3060, see Supplementary Movies 1) and high accuracy (on test data, MAE: 4.19 K, MAPE: 0.49%; on training data, MAE: 4.19 K, MAPE: 0.48%; the standard deviation of the total data points (800×1000×39×13) is 181.7 K). In contrast, the conventional DNS model costs about 8 h with 4 CPUs (Intel Xeon E5-2620, 2.40 GHz, 32 G RAM for each CPU) in parallel (Intel MPI) to complete the DNS process.

### Adaptive adjustment of the secondary cooling

The secondary cooling is an important factor affecting metallurgical length (the distance where solidification is sufficiently complete in slab centre, calculated based upon solid fraction along the slab direction as shown in Fig. 3a), which is a crucial processing variable used to estimate the casting defects distributed along the centerline of the slab, such as centerline segregation, porosity, inclusions, alloy-rich regions, and even cracks. They are especially harmful in rolling process of the highly alloyed steel slabs^30,31^. Robust and accurate control of secondary cooling is vital to prevent or even suppress the defects and to the produce high-quality steel slabs. Real-time control of secondary cooling to control the metallurgical length is highly desirable to meet the demands of product quality and operational safety. Thus far we can predict the temperature field on millisecond timescale, the ReP model can provide the information for the adaptive real-time closed loop process control. To narrow down the enormous search space during adaptive adjustment, we further combine the quick prediction model with Bayesian optimisation (BO)^32^ to solve when one or more technological parameters (casting speed and cooling water flow rate in eight different cooling zone) change, and interrogate how to set other parameters to keep the metallurgical length.

**Fig. 4:**
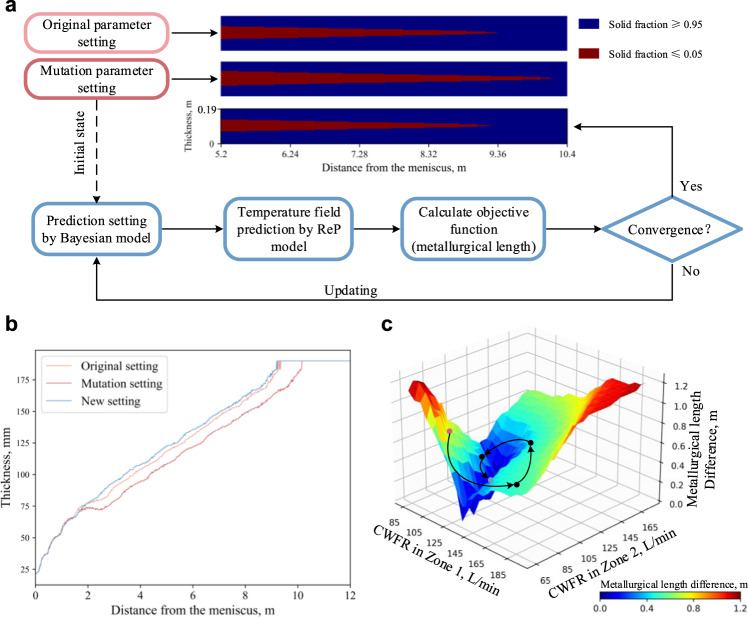
Schematic of secondary cooling water optimisation using ReP model and Bayesian optimisation. **a** Workflow of the secondary cooling water optimisation. When the parameter setting changes, the mutation state is input to the Bayesian model as the initial state to obtain the optimised setting; then the new setting is used in the ReP model to predict the temperature field; finally, calculate the objective function to determinate whether to stop the optimisation search or not. **b** Comparison of the shell thickness curve under original setting (Orange curve), mutation setting (Red curve) and optimised setting (Blue curve). **c** Metallurgical length difference under the corresponding parameters (cooling water flow rate (CWFR)) mapped with ReP model. Points are scattered to show the search path of the BO process (Red points correspond to the start point, black point correspond to the intermediate point and blue point correspond to the end point).

We randomly choose a predicted result as the initial state, and increase the casting speed from 1.3 m min^−1^ to 1.37 m min^−1^. The metallurgical length is lengthened from 9.36 m to 10.18 m. Then, we use the BO to search for the best CWFR settings for Zone 1 and Zone 2 to minimise the change of the metallurgical length. The BO will provide a new prediction parameter setting based on Gaussian process. Then the new parameter setting is input to our ReP model to predict the 3D temperature field. The next step is to calculate the objective function and iterate the Bayesian model as shown in Fig. 4a. The original, mutation and new parameter setting are listed in Table 1. Here, we use the difference of the metallurgical length as the objective function, and the solidification state is calculated according to the temperature field by Eq. 8, where *f*_L_, *T*, *T*_Solidus_ and *T*_Liquidus_ are liquid fraction, temperature (K), solidus temperature (1715 K), and liquidus temperature (1786 K), respectively. In this experiment, it takes BO 22 iterations to converge, and a new parameter setting leading to the same metallurgical length is found. The comparison of the shell thickness under the original, mutation and new parameter settings is shown in Fig. 4b. The thickness gets thinner with the increase in casting speed, and besides the BO finds a higher CWFR in Zone 1 and Zone 2 arrangements to get a 9.32 m metallurgical length, which is similar to the original one. A metallurgical length difference map is shown in Fig. 4c, and some points during the BO iterative process are drawn to show the searching path. The map shows that the lowest difference locates in an arc range. This corresponds to the fact that the cooling water flow rate in Zone 1 and Zone 2 should be complementary, and either too low or too high CWFR will result in a larger offset. With the help of our ReP model, this optimisation process only takes 5.2 s, while it would be days for the conventional DNS.

8 $${f}_{{{{{{\rm{L}}}}}}}=\left\{\begin{array}{cc}0 & T\le {T}_{{{{{{\rm{S}}}}}}{{{{{\rm{olidus}}}}}}}\\ \frac{{T-T}_{{{{{{\rm{S}}}}}}{{{{{\rm{olidus}}}}}}}}{{T}_{{{{{{\rm{L}}}}}}{{{{{\rm{iquidus}}}}}}}-{T}_{{{{{{\rm{S}}}}}}{{{{{\rm{olidus}}}}}}}} & {T}_{{{{{{\rm{S}}}}}}{{{{{\rm{olidus}}}}}}} < T < {T}_{{{{{{\rm{L}}}}}}{{{{{\rm{iquidus}}}}}}}\\ 1 & T\ge {T}_{{{{{{\rm{L}}}}}}{{{{{\rm{iquidus}}}}}}}\end{array}\,\right.$$

**Table 1:** The original, mutation and new parameter setting during the optimisation.

		Original	Mutation	New
Casting speed (m min^−1^)		1.3	1.37	1.37
Cooling water flow rate in each cooling zone (L min^−1^)	Zone 1	102	102	**127**
	Zone 2	87	87	**106**
	Zone 3	89	89	89
	Zone 4	85	85	85
	Zone 5	70	70	70
	Zone 6	30	30	30
	Zone 7	77	77	77
	Zone 8	49	49	49

Bold font indicates the parameter that has changed.

**Fig. 5:**
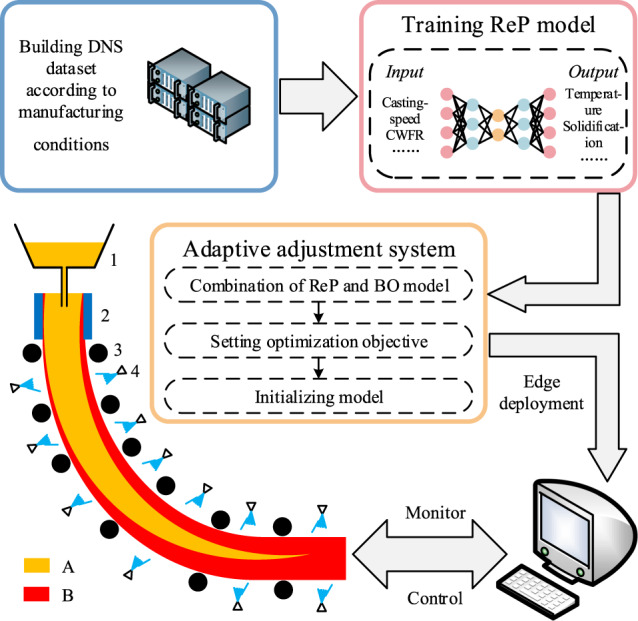
Schematic of intelligence casting. First, we need to build DNS dataset to train the ReP model. Then, the ReP model can be combined with BO model to form the adaptive adjustment system, after setting the optimisation objective and initialising the model, the system can be deployed at the front of the manufacturing factory to control the CC process. 1: Tundish. 2: Mold. 3: Roll support. 4: Spray nozzle. A: Liquid metal. B: Solidified metal.

The experiment above uses a target metallurgical length as the objective function. Moreover, other objective functions, such as target temperature distribution, target shell thickness, and target temperature at a certain position, can be set for technological parameter searching to achieve different quality objectives and even a hybrid one. In addition, other optimisation regulation algorithms, such as simulated annealing, differential evolution, and particle swarm optimisation, can also be combined with our model. Only a few seconds are needed for the ReP model to run hundreds of iterations for the optimisation regulation algorithms and to ensure the convergence. So it is suitable to deal with the complex changes and requirements in real manufacturing environment. Besides, our well-trained ReP model is more deployment friendly and can be performed on a regular personal computer. As shown in Fig. 5, the training of the ReP model is a one-time cost, the low computing time and cost makes the adaptive adjustment system have the advantages of edge deployment and control, and realised intelligence casting to improve the steel quality and reduce costs.

### Dataset volume analysis

As a data-driven model, the performance and robustness of DL model are very sensitive to the datasets size, and small size datasets impose great challenge in developing DL model. In this study, we simulated 700 CC DNS temperature field data to train our DL model and achieved satisfactory performance. In order to investigate the impact of dataset size on model performance and explore ways to improve, we first illustrate the effect of data volume on model performance by analysing the relationships between training data and test data, and then compare the test results under different training data volumes.

**Fig. 6:**
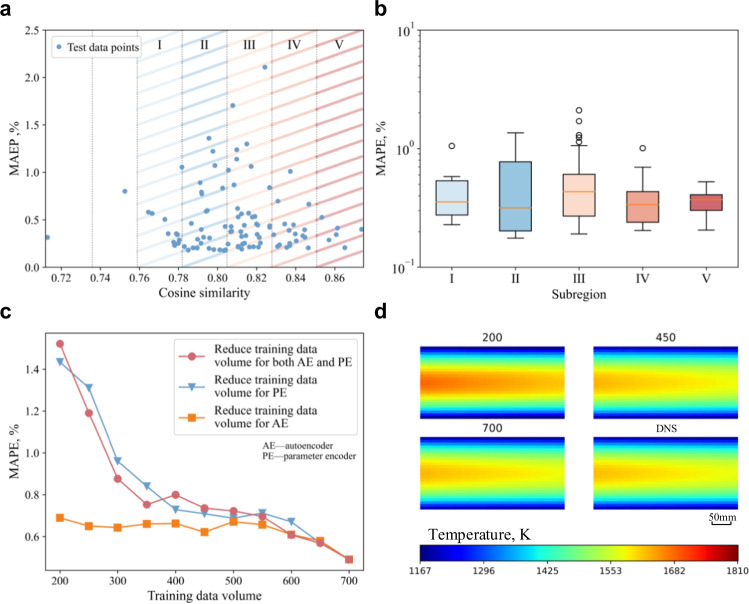
Experiment results: the effect of training data volume on the prediction results. **a** Mean absolute percent error (MAPE) distribution of 100 test data. The cosine similarity between the test technological parameter set and the whole training technological parameter sets is set as the *x*-axis. Shadings indicate different sub-regions. **b** Box-whisker plot for five sub-regions I–V in **a**, the number of independent samples for box I–V are 11, 25, 38, 18, 6, respectively. **c** MAPE results when reducing the training data volume from 700 to 200. Red circle curve: reduce the training data volume of both the autoencoder and parameter encoder; blue triangle curve: reduce the training data volume of the parameter encoder and keep 700 training data of the autoencoder; yellow square curve: reduce the training data volume of the autoencoder and keep 700 training data of the parameter encoder. **d** Comparison of prediction results for different training data volume and DNS result.

In Fig. 6a, we show the MAPE distribution of 100 test data, where the cosine similarity between the test technological parameter setting and the whole training technological parameter setting is set as the x-axis. Higher cosine similarity means that this test data is closer to the coverage of the training data, which should result in better performance on this test data. For a better view, we divide the region in Fig. 6a into seven sub-regions equally and draw box-whisker plots for the last five sub-regions in Fig. 6b, we can see the trend of MAPE decreasing with the increase in cosine similarity. Besides, we compare the MAPE results when we reduce the training data volume from 700 to 200, as shown in Fig. 6c. Obviously, the MAPE results deteriorate as the training data volume reduces. Due to the too many (nine) technological parameters and the too large sampling space, our training data cannot cover the entire sampling space, so it is hard to map the relationships between the technological parameter sets and the latent code perfectly. Increasing the data volume to cover more sampling space should improve the performance. But we find a diminishing marginal effect when changing dataset volume, as shown in Fig. 6c. Therefore, the amount of data to achieve a perfect result might be rather extensive. When the amount of data is limited, the performance and robustness of the model can be improved through data augmentation^33^, regularisation^34^, transfer learning^35^, and knowledge distillation^36^.

However, when comparing the results of reducing the training data volume of the autoencoder and reducing the training data volume of the parameter encoder (when reducing the training data volume of one model, the training data volume for another model stays at 700). The MAPE curve is similar between the red circle curve (reducing the training data volume of both the autoencoder and parameter encoder) and the blue triangle curve (reducing the training data volume of the parameter encoder), but the MAPE results do not deteriorate much when only reducing the training data volume of the autoencoder (yellow square curve). It can be inferred that the bottleneck of our ReP model is the parameter encoder, and the autoencoder can be well-trained with only a small amount of data. The loss curve also shows that the parameter encoder is a little bit underfitting while the autoencoder fits well (see Supplementary Figs. 7 and 8). Figure 6d shows the comparison of ReP results by different training data volumes. Clearly, the ReP result is improved with the increase in the number of training data.

## Discussion

In this study, we developed a ReP model to predict the 3D CC temperature field with high precision and throughput. Proving that the DL technology can learn the relationships between the DNS data and the corresponding technological parameter setting, and then make accurate predictions. The final performance and accuracy of the Rep model depends on two aspects: accuracy of the numerical simulation and completeness of dataset space; and the accuracy of DL model. Therefore, to make more precise prediction, a better solidification model is essential. As a first attempt for this approach, the main purpose of this study is to verify the feasibility of the approach, so we do not give much consideration to the CC DNS dataset, and current dataset contains only the temperature information of the CC. In fact, when we use the most advanced DNS models (such as macrosegregation model and dendritic structure model) to generate datasets, these CC DNS datasets will contain more valuable information (process variables), and we can fully develop ReP models for macrosegregation and dendritic structure prediction, which is of great significance for the quality and intelligent control of CC process, but lots of efforts and times may be required to build this kind of dataset. In addition, this approach is content independent in some ways, so it is possible to be spread to other research areas to actualise similar ReP processes to help accelerate scientific research.

Furthermore, we take the advantages of the ReP model and combine it with optimisation regulation algorithms to achieve fast adaptive adjustment. In our model, the adaptive adjustment is completed in seconds, which is much faster than using conventional DNS technology, demonstrating the potential application scenarios and capabilities. The trained ReP model does not require extensive computing resources as the conventional DNS, which is more computational friendly for edge deployment and computing. Thus, it is also possible to deploy the ReP model at the front of the fabrication to help implementation fast adaptive adjustment to improve process control as shown in Fig. 5.

Besides, it is necessary to discuss the limitations and shortcomings of our model as well:

- As a data-driven method, the dataset is a common concern for researchers. Although a lot of effort has been made to produce a large number of the CC dataset, our ReP model has yet reached a perfect accuracy. As illustrated in Fig. 6c, the dataset, especially for parameter encoder, needed to be further enriched to achieve higher accuracy of the trained ReP model. Moreover, this work provides the most complete dataset to digitalise the metallurgical process.
- The ReP model can only predict the results within the training data space. For example, current ReP model is difficult to accurately predict the CC results with a casting speed larger than 1.65 m min^−1^, because the operating parameter is beyond the range of the operating conditions (0.75–1.65 m min^−1^) in the training data. We used the ReP model to predict the temperature at the end of the slab under the extreme casting process conditions, i.e. the minimum casting speed and maximum cooling water flow, and obtained a minimum temperature of 598 K, but the actual value should be 372 K from DNS result. The reason is that the minimum temperature in our dataset is 604 K, and the predicted minimum temperature will be limited by the training dataset and not much lower than the lowest temperature in this dataset. Therefore, to obtain higher prediction accuracy, it is necessary not only to increase the dataset, but also to expand the range of processing processes and resultant temperatures as much as possible.

More work is needed to completely replace the conventional DNS. Having said that our model has excellent advantages in terms of computational speed and resources, and it is accurate enough under the conditions investigated. The digitalisation tool proposed here is therefore valuable for accelerating the manufacturing science research and technology take-up.

## Methods

### Data preparation and preprocessing

In this study, we developed a combined hybrid 3D/2D model for heat transfer, fluid flow, and solidification simulation using computational fluid dynamics (CFD) calculation, according to the reference^3^ to generate our dataset (see Supplementary Figs. 1 and 2, Supplementary Tables 1–3 and Supplementary Note 1). The solution of the CC model is performed on the ANSYS FLUENT 14.5 CFD software. We chose this model for three reasons. First, it is a verified model that can provide reliable results. Second, the 3D simulation results are more representative than the 2D simulation ones. Third, the computational speed is much faster than a 3D simulation model, which is a very important consideration because a great number of simulations need to be performed for big datasets. This numerical model divided the computational domain into two parts—the 3D turbulent flow region and the 2D laminar flow region. The velocity of the molten steel in the casting direction is equal to the casting speed after the 3D turbulent flow region and 2D laminar flow region interface (which is set at the end of Zone 3) and thus forming plug flow. Moreover, as reported^37^, the heat flux in the casting direction accounts for just a little (about 3–6%) of the total heat loss. These phenomena enable us to ignore the heat flux in the casting direction in the laminar flow region. In addition, due to the heat transfer, cooling condition, and solidification process of slab continuous casting are of good symmetry in the width and thickness directions of the slab, the difference in cooling intensity between the inner and outer wide surfaces of the slab, and the effect of the bending and straightening process on the thermal contraction deformation, can be neglected to reduce the computational cost^38^. One-quarter of the strand was included in the computational domain. The computation cost has obviously reduced, providing us an advantage for generating a large amount of data. We employed this CC model to produce our dataset under different casting speeds and cooling water flow rates. In addition, this model considers the uneven distribution of cooling water in the wide face direction according to the actual process, and there are eight secondary cooling zones with different cooling water rates.

The DNS model produced the 20362×765×95 mm^3^ temperature field (a quarter of the slab). In other words, a data dimension of 1000×39×13. However, this data shape was too large for a DL model. In order to reduce the computational cost, we divided the data into 13 layers in the thickness direction (*Z*-direction) as illustrated in Fig. 1. Moreover, we zero pad the data in the width direction (Y-direction) to shape 1000×40×1 and normalise them between 0 and 1 to simply the scaling operation. We used Latin hypercube sampling (LHS)^39^ to collect our sampling points to guarantee the randomness and uniformity of the dataset. The sampling range for each parameter is listed in Supplementary Table 4. 3D DNS data of 800 model outputs was calculated, and we divided the dataset into 700 dataset for training and 100 dataset for testing. Then we expanded the training data through data augmentation^33^. The test data were only used to test the model performance, instead of participated in any training processes.

### Real-time prediction model

The framework of the whole ReP model is shown in Fig. 1, the backbone of our autoencoder and parameter encoder is the ConvNeXt block and Mixer layer, which are built according to the corresponding references^28,40^ (see Supplementary Fig. 6). The Down/Up-sample layer is a 2×2 Conv2D/Conv2DTranspose with strides 2 to half/double the height and width of the feature map. The projection layer is a 1×1 Conv2D layer to change the dimension of the feature map. MLP layer is placed at the end of the encoder and the beginning of the decoder as a projection head to operate on one-dimension data, and is very important for extracting the latent code. A detailed structure is listed in Supplementary Table 5 and 6. The reason for separating the whole ReP model into autoencoder and parameter encoder is to introduce data augmentation to enhance the robustness of the model (see Supplementary Fig. 9). Adam optimiser^41^ was used for both two DL models at learning rates of 0.0013 and 0.01, respectively. Early stopping^42^ and learning rate decay^43^ were adopted to speed up the training progress. We set a 0.9 learning rate decay for five epoch patience and found it beneficial for training. The activation function and loss function for both two DL models were gelu^44^ and MAE. The batch size for the autoencoder and parameter encoder is 32 and 128, respectively.

All DL models were trained on NVIDIA HGX A100 40GB GPU using the Tensorflow^45^ library, and it took about seven hours to train the ReP model (about seven hours for the autoencoder and 10 min for the parameter encoder).

### Bayesian optimisation

As a framework for global optimisation of expensive-to-evaluate black-box functions, BO has become popular due to its remarkable performance in hyperparameter tuning of machine learning algorithms recently. The goal of Bayesian optimisation is to build a distribution based on previous measurements, priori information, using a Gaussian random process. It has been invented and used for a long time^46^ and constantly upgraded and evolved^32,47^. In this work, a regular version of BO is achieved using the bayes_opt^48^ library in Python, an efficient implementation of the BO methodology for nonlinear optimisation, experimental design and hyperparameter tuning. The initial step of random exploration is set to 5. With expected improvement function as acquisition function and set an exploration ratio of 0.1.

### Supplementary information

Supplementary Information

Description of Additional Supplementary Files

Supplementary Data 1

Supplementary Movie 1

## Data Availability

Representative research data are given in the figures (and Supplementary Data). The source data for Fig. 3a–c is provided as Supplementary Data 1. The unedited raw data generated by Fluent that makes up the key dataset is accessible in figshare (10.6084/m9.figshare.22810319.v1). Other generated and/or analysed datasets that support the findings of this study are available from the corresponding author upon reasonable request.

## Code availability

All custom code used in this work, including that used to train and test ReP models, and perform secondary cooling water optimisation with BO, can be obtained from the following publicly accessible GitHub page: https://github.com/LarkMi/ReP.
