# Supplementary material for: Real-time prediction and adaptive adjustment of continuous casting based on deep learning
Source: Commun Eng. 2023 Jun 7;2:34. doi: 10.1038/s44172-023-00084-1 (PMC10955886; doi:10.1038/s44172-023-00084-1)
Supplement: Supplementary file 1 — Supplementary Information [file 44172_2023_84_MOESM1_ESM.pdf]

Supplementary Information for:

Real-time prediction and adaptive adjustment of continuous casting based on deep learning

Ziqing Lu[1], Neng Ren[1], Xiaowei Xu[1], Jun Li[1,*], Chinnapat Panwisawas[2], Mingxu Xia[1], Hongbiao Dong[3], Eric Tsang[4], Jianguo Li[1]

[1]Shanghai Key Laboratory of Advanced High-temperature Materials and Precision Forming, School of Material Science and Engineering, Shanghai Jiao Tong University, Shanghai 200240, China.

[2]School of Engineering and Materials Science, Queen Mary University of London, London E1 4NS, United Kingdom.

[3]School of Engineering, University of Leicester, Leicester LE1 7RH, United Kingdom

[4]School of Computer Science and Engineering, Macau University of Science and Technology, Taipa, Macau, China

Corresponding author: Jun Li (li.jun@sjtu.edu.cn, https://orcid.org/0000-0003-4915-0336)

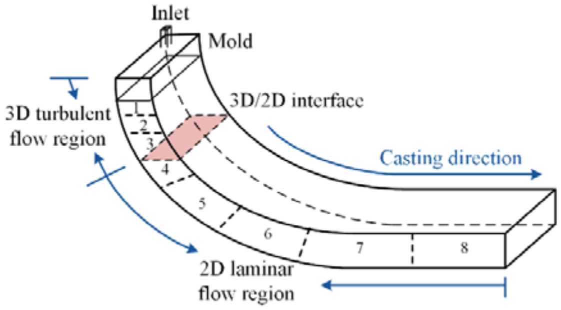

Supplementary Figure 1. Schematic of the continuous casting numerical simulation model. The computation domain is divided into two parts: 3D turbulent flow region and 2D laminar flow region. Mold, 3D/2D interface, and eight different secondary cooling zone are highlighted.

Supplementary Note 1. Key assumptions of our continuous casting numerical simulation model.

1. Since the heat transfer, cooling condition, and solidification process of slab continuous casting show good symmetry in the width and thickness directions, it is acceptable to neglect the difference in cooling intensity between the inner and outer wide surfaces of the slab, as well as the effect of the bending and straightening process on the thermal contraction deformation. Thus, one-quarter of the strand was included in the computational domain to reduce the computational cost.
2. The computational domain is divided into two parts—the 3D turbulent region and the 2D laminar region according to the flow velocity. The velocity of the molten steel in the casting direction is equal to the casting speed after the 3D turbulent flow region and 2D laminar flow region interface (which is set at the end of Zone 3) and thus forming plug flow. Moreover, the heat flux in the casting direction accounts for just a little (about 3%-6%) of the total heat loss. These enable us to ignore the heat flux in the casting direction in the laminar flow region. Therefore, we can reduce the computation costs during the establishment of the dataset.

3. The mushy zone (partially solidified region) is regarded as a porous medium, the porosity of which is defined as the function of liquid fraction.
4. The density of steel is constant, but the specific heat capacity and the heat conductivity of steel are the temperature-dependent properties.
5. Liquid steel is assumed as a Newtonian incompressible fluid.
6. The strand curvature, bulging, oscillation, mold taper, mold oscillation, effect of segregation, and air gap were neglected.

Supplementary Table 1. The geometry and working parameters of the continuous casting process. SEN: submerged entry nozzle.

Parameters	Values
Mold section	1530×190 mm^2
Mold length	800 mm
Inside size of SEN	86×45 mm^2
Outside size of SEN	141×100 mm^2
Port size of SEN	45×73 mm^2
Port angle	-15 degrees
Casting temperature	1811 K
Steel grade	Q345

Supplementary Table 2. Physical properties of steel Q345.

Physical Properties	Values
Density, kg·m^{-3}	7330
Specific heat, J·(kg·K)$^{-1}$	319.59+0.1934×T(K)
Liquidus, K	1786
Latent heat, J·kg^{-1}	25550T0
Viscosity, kg· (m·s)$^{-1}$	0.0062
Thermal conductivity, W·(m·K)$^{-1}$	57.524-0.0164×T(K)
Solidus, K	1715

Supplementary Table 3. Boundary condition. Where L_z, V_c, T_w, w, T_0, σ, ε, T_e and α are the distance from the meniscus (m), casting speed (m/min), slab surface temperature (K), spray water impingement density ($L \cdot m^{-2} \cdot s^{-1}$), water temperature (K), Stefan-Boltzmann constant (5.67×10-8 W/m^{2}K^4), emissivity of the steel (0.8), environmental temperature (K) and angle to the arc of the roll contact in degree, respectively.

Mold surface	$q_m = 2680000 - 276000 \times \sqrt{\frac{60 \times L_z}{v_c}}$
Water spray cooling	$q_s = (2950.190 \times T_w^{-0.235} \times w^{0.805}) \times (T_w - T_0)$
Radiation cooling	$q_{rad} = \sigma \cdot \varepsilon \times (T_w^4 - T_e^4)$

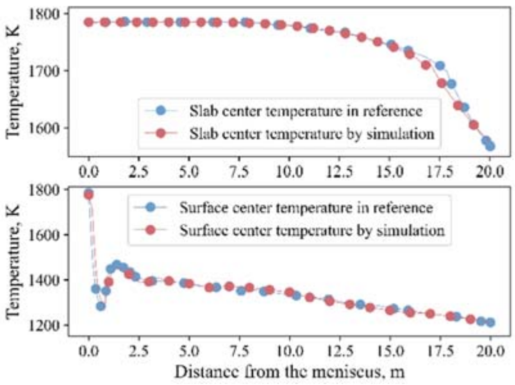

Supplementary Figure 2. Temperature comparison between our computational fluid dynamics simulation result and the reported in reference[1], shows good agreement.

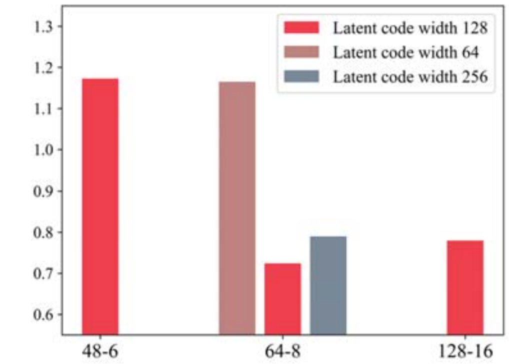

Supplementary Figure 3. Autoencoder structure test for different feature map dimensions and different latent code widths. The best MATE result is 0.72 K, the corresponding structure is to compress the feature map dimension from 64 to 8 and then to a 128×1 latent code. The MATE results get worse to 1.17 K when the feature map dimension reduces to 48 to 6 but only change a little to 0.78 K when the feature map dimension doubles. When the latent code width changes under the 64 to 8 structure, 64 and 256 latent code widths both are significantly worse than the 128 latent code width. The above results demonstrate that the current autoencoder structure is both efficient and effective.

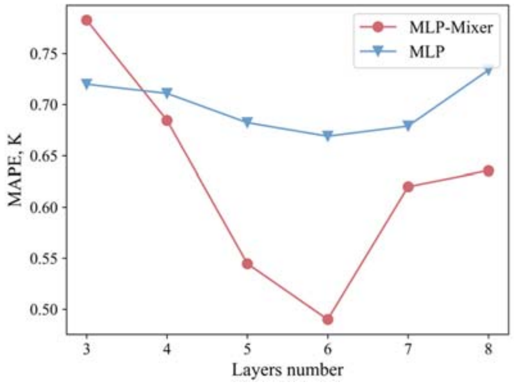

Supplementary Figure 4. Parameter encoder structure test for different layer number and different backbone network. We tested different layer numbers for the MLP parameter encoder and the MLP-Mixer parameter encoder. The MLP structure parameter encoder's inputs are nine technological

parameters plus a position parameter (since we split the 3D temperature field into 13 layers for the autoencoder, so the position parameter stands for the layer information), so the information between layers is not considered in the MLP structure parameter encoder. The best results for MLP and MLP-Mixer parameter encoder are 0.67% and 0.49%, demonstrating the effectiveness of introducing the information between layers by employing an MLP-Mixer parameter encoder.

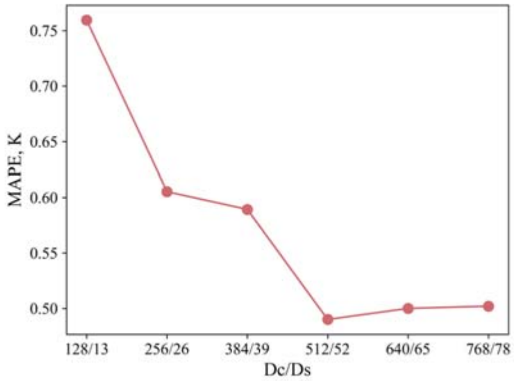

Supplementary Figure 5. Parameter encoder structure test for different Dc/Ds setting, the MAPE results reached 0.49% when we set Ds/Dc as 512/52.

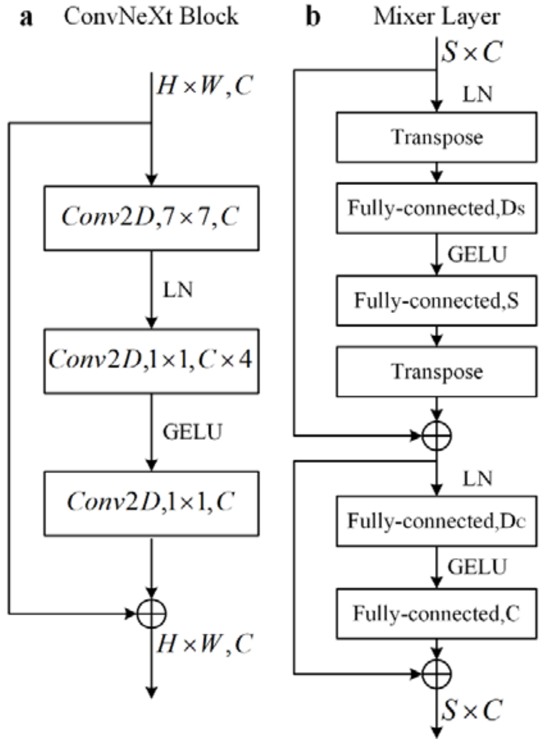

Supplementary Figure 6. Structure of the (a) ConvNeXt Block and the (b) Mixer Layer employed in our real-time prediction model.

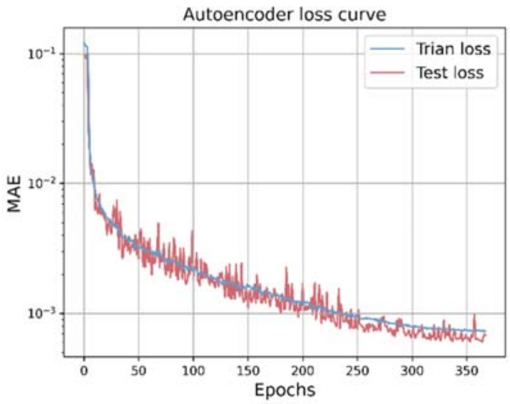

Supplementary Figure 7. An example of autoencoder's loss curve. The training loss is higher than the test loss because we used data augmentation to enhance the training data.

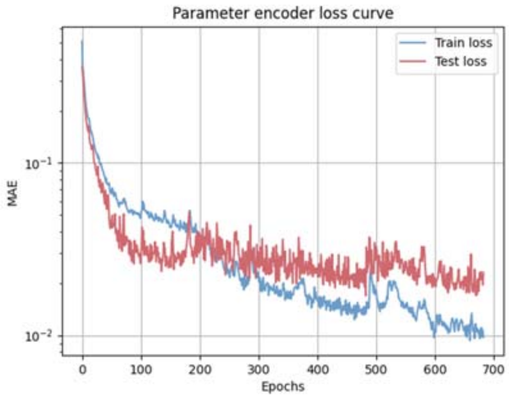

Supplementary Figure 8. An example of parameter encoder's loss curve.

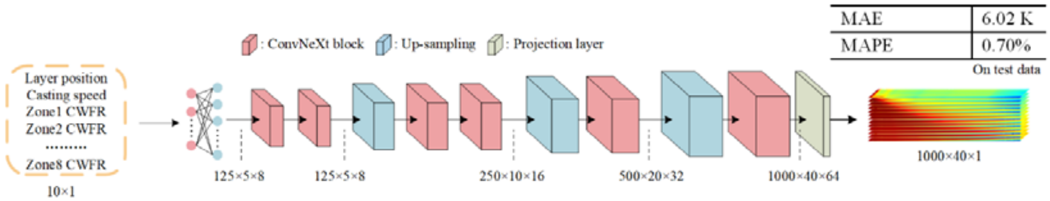

Supplementary Figure 9. Schematic diagram of the structure and results of single model.

Supplementary Table 4. Sampling range of different technological parameters.

Technological Parameters	Casting Speed(m/min)	Cooling Water Flow Rate (L/min)							
		Zone1	Zone2	Zone3	Zone4	Zone5	Zone6	Zone7	Zone8
Min	0.75	0	0	0	0	0	0	0	0
Max	1.65	220	180	160	160	160	160	160	160

Supplementary Table 5. Detail structure of the autoencoder.

Autoencoder		
Encoder	Input Output<br>Output Input	Decoder
Conv2D	1000×40×1<br>1000×40×64	Conv2D
ConvNeXt Block	1000×40×64	ConvNeXt Block
Conv2D	1000×40×64<br>500×20×32	Conv2DTranspose
ConvNeXt Block	500×20×32	ConvNeXt Block
Conv2D	500×20×32<br>250×10×16	Conv2DTranspose
ConvNeXt Block	250×10×16	ConvNeXt Block
ConvNeXt Block	250×10×16	ConvNeXt Block
Conv2D	250×10×16<br>125×5×8	Conv2DTranspose
ConvNeXt Block	125×5×8	ConvNeXt Block
ConvNeXt Block	125×5×8	ConvNeXt Block
Flatten	125×5×8<br>5000×1	Reshape
Fully-connected	5000×1<br>256×1	Fully-connected
Fully-connected	256×1<br>128×1	Fully-connected
Parameters	3.38 M	
FLOPs	40.89 G	

Supplementary Table 6. Detail structure of the parameter encoder.

Parameter encoder	
Layers	Output shape
Fully-connected	9×13
Transpose	13×9
Fully-connected	13×128
Transpose	128×13
Mixer Layer	128×13
Mixer Layer	128×13
Mixer Layer	128×13
Mixer Layer	128×13
Mixer Layer	128×13
Mixer Layer	128×13
Parameters	0.8 M
FLOPs	1.59 M

Reference:

1. M. J. Long, et al.A Combined Hybrid 3-D/2-D Model for Flow and Solidification Prediction during Slab Continuous Casting.Metals.**8**.10.(2018).
