# Supplementary material for: Real-time prediction and adaptive adjustment of continuous casting based on deep learning
Source: Commun Eng. 2023 Jun 7;2:34. doi: 10.1038/s44172-023-00084-1 (PMC10955886; doi:10.1038/s44172-023-00084-1)
Supplement: Supplementary file 2 — Description of Additional Supplementary Files [file 44172_2023_84_MOESM2_ESM.pdf]

Description of Additional Supplementary Files

File name: Supplementary Data 1
Description: Source data for Fig. 3a, Fig. 3b and Fig. 3c

File name: Supplementary Movie 1
Description: Display movie of the real-time prediction model for continues casting
